# Salivary and crevicular fluid interleukins in gingivitis

**DOI:** 10.4317/jced.51403

**Published:** 2014-04-01

**Authors:** Montserrat Boronat-Catalá, Montserrat Catalá-Pizarro, José V. Bagán Sebastián

**Affiliations:** 1 Graduate in Dentistry; 2 Full Professor of Pediatric Dentistry at the University of Valencia; 3 Full Professor of Oral Medicine at the University of Valencia

## Abstract

Introduction: Gingivitis is a frequent inflammatory process of the gum tissue that is mainly caused by the accumulation of plaque. The immune response against inflammatory processes is regulated in part by cytokines. Aims: Given that a continuous inflammation exists in gingivitis, it would be logical to assume that the interleukins will be altered locally in those patients. Therefore, the aim of this review was to check whether there is evidence that the interleukins can be used as diagnostic markers of inflammation levels in patients with gingivitis. Materials and Methods: A bibliographical search was undertaken using the key words interleukin and gingivitis in Pubmed, Cochrane, Scopus and Embase. Only those articles published over the last 10 years that were systematic reviews, case-controls or cohort studies in which interleukins in saliva and/or crevicular fluid was investigated in patients with gingivitis were selected. Results: Finally 15 articles were selected, all of them being case-control studies. The interleukins analyzed in the reviewed articles were: IL-1β, IL-8, IL-18, IL-11, IL-12, TNFα, IL-4, IL-17, IL-1α and IL-6. The most commonly studied interleukin is IL-1β and most authors agree that it is higher in the saliva and/or crevicular fluid of patients with gingivitis. Therefore, it could be used as a diagnostic marker of the degree of inflammation in gingivitis. Moreover, as far as the other interleukins studied are concerned, there is no clear consensus among the authors. Conclusion: There is sufficient evidence to suggest that IL-1β in saliva and/or crevicular fluid can be used as a marker of the degree of inflammation in gingivitis.

** Key words:**Interleukins, gingivitis, saliva, crevicular fluid.

## Introduction

Gingivitis or inflammation of the gum tissue is of a wide ranging etiology in which local factors intervene such as dental plaque, calculus (tartar), food impaction, irritation from restorations, etc. and several systematic factors such as pregnancy, diabetes, nutritional alterations, etc. (1)

The most common form of gingivitis is that caused by bacterial plaque, which is the inflammation of gum tissue as a result of the bacteria located on the gingival margin (2). The relationship between bacterial plaque and gingival inflammation has frequently been postulated as the cause of gingivitis and its role in the etiology has been confirmed in experimental gingivitis studies on human beings (3).

The immune system is the barrier against infectious microorganisms that affect the oral cavity (4). The complex network of cytokines that intervene in the immune response of the host against external attacks include pro-inflammatory cytokines, anti-inflammatory cytokines and receptor cells for these cytokines (5).

Interleukins are cytokines which play an important role in communication between inflammatory cells following the activation of the immune system (6). It would, therefore, be of great interest to know which of them act as mediators in gingival inflammation.

In gingivitis there is a build up of bacteria that causes inflammation of the gum tissue and, therefore, triggers the innate immunological response, an abundant series of macrophage cells, in turn, producing interleukins. If there is continuous inflammation in the gingivitis, it is understood that the interleukins will be altered in patients with this pathology.

The main aim, therefore, of this review was to find out from the literature available the degree to which interleukins can be used as diagnostic markers of the degree of inflammation in gingivitis.

The specific objectives were:

1. To establish which interleukins show a diagnostic correlation with gingivitis.

2. To show the degrees of scientific evidence and their grades of recommendation, following the criteria of the Oxford Centre for Evidence-Based Medicine Levels of Evidence 2010, for the various interleukins analyzed.

## Material and Methods

A bibliographical search was undertaken using the key words: “interleukin and gingivitis” in Pubmed, the cutoff point for that search being articles published over the last 10 years. This was then supplemented by a search using the same key words in the following databases: Cochrane, Scopus and Embase.

The inclusion criteria for selecting articles were as follows:

1. Articles had to be systematic reviews, case-control studies or cohort studies.

2. The articles had to study interleukins in saliva and/or crevicular fluid in humans.

3. The samples had to be made up of individuals with gingivitis.

Finally 15 articles were selected that were then analyzed from the perspective of variables such as: the interleukin analyzed, the type of study, the laboratory method, the sample, the type of patient selection, the results, the level of evidence and grade of recommendation for each article.

## Results

- Data on the articles selected

**Figure 1:**
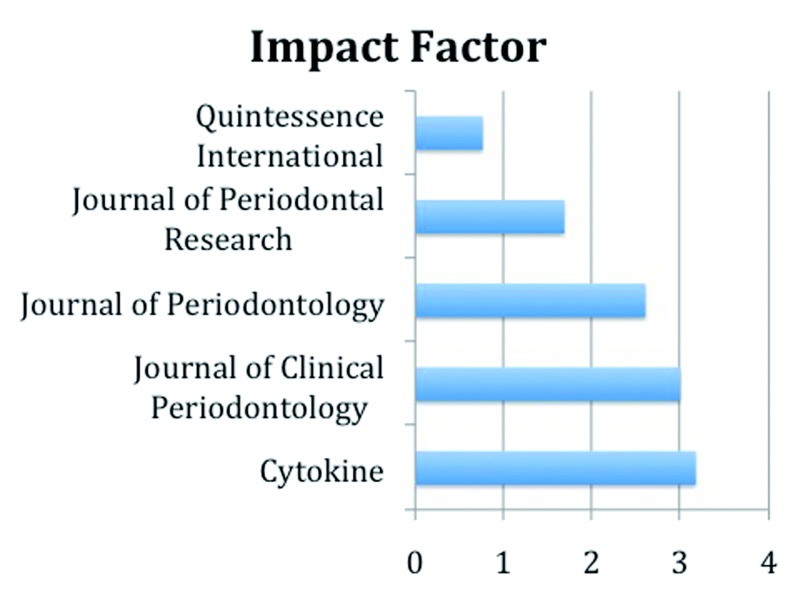
Impact factor of analyzed articles.

All the articles selected were case-control studies. The oldest article was from 2003 and the most recent from 2013. The articles came from 8 different countries mostly from Turkey, India and the United Kingdom. Most articles were obtained from the Journal of Periodontal Research, the Journal of Periodontology and the Journal of Clinical Periodontology. Finally, the mean impact factor of the journals from which the articles came was 2.243. (Fig. 1)

The total number of patients included in the samples from all the articles amounted to 743, with a mean age of 32.70 years. The percentages per gender of the total sample were 43.92% for males and 56.08% for females.

- Data on findings in interleukins

The relationship of interleukins in saliva and crevicular fluid with gingivitis was initially analyzed in 2003 by Faizuddin *et al*.(7), who undertook a case-control study in which IL-1β in the crevicular fluid of patients was analyzed. In this study their results showed that the levels of IL-1β in the crevicular fluid of patients with periodontitis were higher than those in patients with gingivitis and also that the levels of this interleukin in gingivitis and periodontitis were much higher than in healthy patients.

Tsai *et al*. in 2005 (8), after carrying out a case-control study in which they analyzed interleukins 12 and 16, found that IL-16 was higher in the crevicular fluid of healthy patients, followed by those with gingivitis and periodontitis. They also found that the total concentration of IL-12 was significantly higher in the crevicular fluid of patients affected by gingivitis and periodontitis than in healthy patients.

Deinzer *et al*. (9), in the year 2007, published a case-control study which compared a group of gingivitis patients with another of experimental gingivitis. Outstanding among their results were that the individuals who were subject to experimental gingivitis presented higher IL-1β and lower IL-8 levels at 4 weeks than individuals with persistent gingivitis.

Yücel *et al*. (5), in 2008, found, in a case-control study of patients with gingivitis and patients with periodontitis, that the amount of IL-11 and IL-1β was higher in the gingivitis group than in the control group. They also found that IL-12 was higher in the group with chronic periodontitis.

That same year Ülker *et al*. (10) published a case-control study consisting of a group of children in which they found that in non-stimulated total saliva, TNFα was higher in healthy children and IL-1β higher in children with gingivitis. Lastly Pradeep *et al*. (6), in their case-control study, found that the highest concentration of IL-4 was in the control group, the lowest in the group with periodontitis, and the group with gingivitis had intermediate concentrations of this interleukin.

In 2009 Türkoglu *et al*. (4), in a case-control study, analyzed IL-18 in the crevicular fluid of patients with periodontitis and gingivitis, but found no differences in the levels of this interleukin in the different study groups. In 2009 Pradeep *et al*. (11) also published a case-control study in which they found that the lowest levels of IL-18 were in the control group patients and the highest levels in patients with periodontitis, with intermediate concentrations of this interleukin in the gingivitis group, and that the levels of IL-17 were imperceptible in all study groups. In a later publication by Pradeep *et al*. (12) the outstanding result obtained was that IL-18 was higher in the crevicular fluid of patients with periodontitis and had a positive correlation with the seriousness of the disease.

Offenbacher *et al*. (13), in 2010, in their case-control study found that in experimental gingivitis, IL-1β and IL1α increased, while IL-8 decreased. In the same year, Trombelli *et al*.(14) found, after undertaking a case-control study on experimental gingivitis, that IL-1β in experimental gingivitis was greater than under normal conditions. Perozini *et al*. (15) analyzed IL-1β in another case-control study and found that it was highest in the periodontitis group, but found no differences between the gingivitis and control group.

Scott *et al*. in 2012 (16) carried out a case-control study in which they also analyzed IL-1β in patients with experimental gingivitis and found that this interleukin increased during experimental gingivitis and then returned to initial levels following the return to normal hygiene. Again in 2012 Becerik *et al*. (17) published a case-control study in which they obtained results showing that the levels of IL-1β and IL-6 in patients with gingivitis were higher than in the control group, although these results were not statistically significant.

Finally, Ertugrul *et al*. (18), in 2013, conducted a case-control study in which they found that IL-8 was higher in patients with gingivitis compared to the control group and also that there is a positive correlation between periodontal parameters and the interleukins: IL-8, IL-1β and TNFα ( Table 1).

**Table 1:**
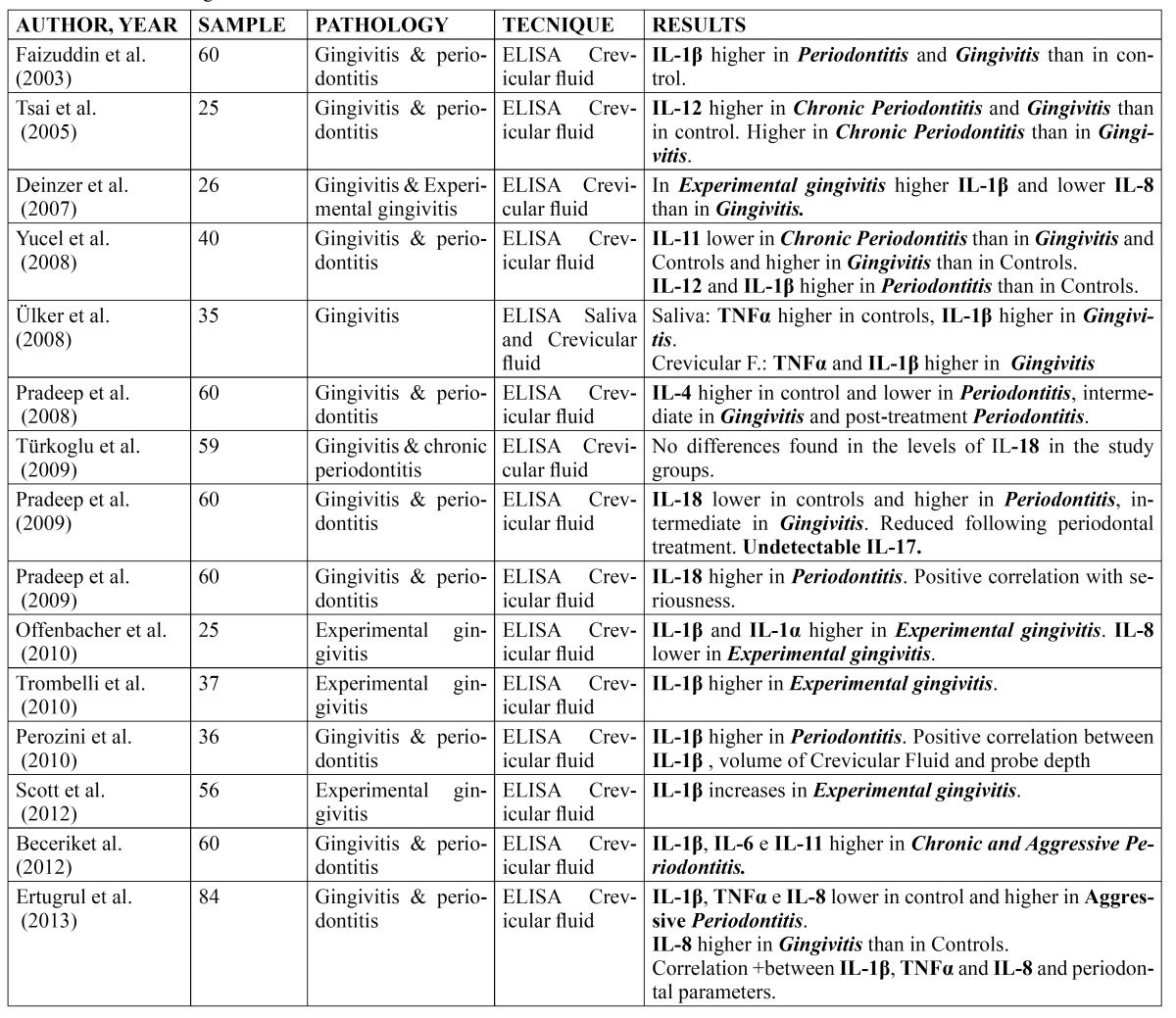
Data on findings in interleukins.

- The articles’ level of evidence and grade of recommendation

Following the criteria of the Oxford Centre for Evidence-Based Medicine Levels of Evidence 2010, all the articles reviewed are case-control studies and correspond to the level of evidence IIIb and a B grade of recommendation.

## Discussion

Having reviewed the literature, we can confirm that IL-1b is the most commonly studied interleukin by authors who have tried to establish inflammation markers in gingivitis. Ten studies have investigated this interleukin, all of them being case-control studies and all analyzing IL-1b in crevicular fluid using the ELISA technique. Some of these studies (9,13,14,16) analyze this interleukin in experimental gingivitis and all found that it is higher in the crevicular fluid of patients with experimental gingivitis and that the levels of this interleukin return to normality following the clinical resolution of the gingivitis (13,16). Three of the studies analyzed this interleukin (5,7,10) in non-experimental gingivitis, the authors finding that IL-1b was higher in patients with gingivitis when compared to those of the control group and that these results were statistically significant. However, in other studies where the results showed that IL-1b was higher in the crevicular fluid of patients with gingivitis, the results were not statistically significant (15,17). Lastly, as far as findings on IL-1b are concerned, one of the studies published only found a positive correlation between the clinical periodontal parameters and the amount of interleukin in crevicular fluid (18). Therefore, the majority of the studies that have analyzed IL-1b in saliva and/or the crevicular fluid of patients with gingivitis are in agreement that this interleukin is a reliable marker of the level of inflammation in gingivitis.

IL-8 is analyzed in three of the studies reviewed (9,13,18). All of these are case-control studies and use the ELISA technique to measure the interleukins. In two of them (9,13) levels of IL-8 in experimental gingivitis are analyzed and both studies observe a decrease in this interleukin in the crevicular fluid of patients with experimental gingivitis. However, the authors explain that their results appear contradictory and that this may be due to the study not being undertaken over a sufficiently long period of time (9), whereas others conclude that inducing gingivitis is related with the suppression of several cytokines such as IL-8 (13). Contrary to these results, there is one study that claims that IL-8 is higher in the crevicular fluid of patients with gingivitis (18). The results found on IL-8 are, therefore, contradictory and do not allow us to classify this interleukin as a marker of inflammation in gingivitis, given that more studies are required to analyze this interleukin in naturally occurring gingivitis rather than in experimental gingivitis.

Three of the case-control studies analyze IL-18 (4,11,12), employing the ELISA technique. Two of these publications (11,12) coincide in finding a lower amount of IL-18 in the control group and a higher amount in the periodontitis group, with intermediate levels for gingivitis. However, the study of Turkoglu *et al*. (4) did not find any differences in the level of this interleukin in the different study groups. There is, therefore, no clear consensus on the usefulness of IL-18 as an inflammation marker in gingivitis.

IL-11 was analyzed in two case-control studies in patients with gingivitis (5,17) using the ELISA technique. Whereas in one of them the authors found a greater amount of IL-11 in the crevicular fluid of patients with gingivitis (5), in the other, they only found a higher amount of this interleukin in the crevicular fluid of patients with periodontitis (17), so more studies are required to analyze this interleukin as, at the moment, there is no clear consensus in terms of its relationship with gingivitis.

Two authors analyzed IL-12 (5,8) in their case-control studies and there is no clear consensus between them, as in one study they found a greater amount of IL-12 in gingivitis and periodontitis than in the control group (8), whereas in the other they only found this interleukin to be related with periodontitis (5).

TNFα is studied in two of the studies analyzed, both being case-control studies and using the ELISA technique to measure the interleukins. Outstanding among the results is that in one of these studies it was found that in non-stimulated saliva TNFα was higher in the control group, even though these results were not statistically significant (10). This study also found that TNFα in crevicular fluid was greater in patients with gingivitis than in the control group (10). Nevertheless, in another publication, the authors only found a positive correlation between the periodontal parameters and TNFα. These two results are difficult to compare as the ages of the samples differ widely and this could affect the results. Therefore, in order to be able to discover whether there is a relationship between TNFα and inflammation in gingivitis further studies are required with groups of similar ages.

Finally, four of the interleukins reviewed are analyzed separately in four different articles and the results the authors found for each one were as followed: IL-4 was found to be highest in the control group and lowest in the periodontitis group, with intermediate levels for gingivitis (6). IL-17 was found to be almost imperceptible in all study groups (11). IL-1α was found to be higher in the crevicular fluid of patients who had been subject to experimental gingivitis (13). Finally, for IL-6, the authors found that in the gingivitis group, IL-6 was higher, but the results were not statistically significant (17).

## Conclusions

Following our analysis of the results of the studies, we can establish the following conclusions.

Firstly, of all the interleukins studied to date in crevicular fluid and/or the saliva of patients with gingivitis, IL-1b has been the most analyzed and that the majority of authors (70%) that have investigated it in their studies agree that it is higher in situations of gingivitis and experimental gingivitis. All these studies are comparable as they are case-control studies and the interleukins are measured using the same technique. Therefore, we can conclude that IL-1b can be used as a diagnostic marker of inflammation in patients with gingivitis.

Secondly, as far as the other interleukins studied are concerned: IL-12, IL-8, IL-11, IL-4, TNFa, IL-18, IL-17, IL-1a, and IL-6, we have not found a consensus among the authors and their results do not coincide sufficiently so as to classify them as diagnostic markers of degrees of inflammation.

Lastly, as the level of evidence of all the articles reviewed in undertaking this work according to the Oxford Centre for Evidence-Based Medicine Levels of evidence 2010 is IIIb and the degree of recommendation of the same is B, we can say that the evidence on using IL-1b in saliva and/or crevicular fluid as a marker of the degree of inflammation in gingivitis is conclusive.
